# Supplementary material for: Skin-electrode iontronic interface for mechanosensing
Source: Nat Commun. 2021 Aug 5;12:4731. doi: 10.1038/s41467-021-24946-4 (PMC8342427; doi:10.1038/s41467-021-24946-4)
Supplement: Supplementary file 1 — Supplementary Information [file 41467_2021_24946_MOESM1_ESM.pdf]

Supplementary Information

Skin-electrode Iontronic Interfaces for Mechanosensing

Pang Zhu[1,#], Huifeng Du[2,#], Xingyu Hou[1,#], Peng Lu[1], Liu Wang[1,2,3], Jun Huang[1], Ningning Bai[1], Zhigang Wu[1,4], Nicholas X. Fang[2,*], Chuan Fei Guo[1,3,*]

[1]Department of Materials Science and Engineering, Southern University of Science and Technology, Shenzhen Guangdong 518055, China

[2]Department of Mechanical Engineering, Massachusetts Institute of Technology, 77 Massachusetts Avenue, Cambridge MA, 02139, USA

[3]Centers for Mechanical Engineering Research and Education at MIT and SUSTech, Southern University of Science and Technology, Shenzhen Guangdong 518055, China

[4]State Key Laboratory of Digital Manufacturing Equipment and Technology, Huazhong University of Science and Technology, Wuhan 430074, China

[#]Pang Zhu, Huifeng Du, and Xingyu Hou contributed equally to this work.

*Correspondence and requests for materials should be addressed to C. F. Guo (email: guocf@sustech.edu.cn) or N. X. Fang (email: nicfang@mit.edu)

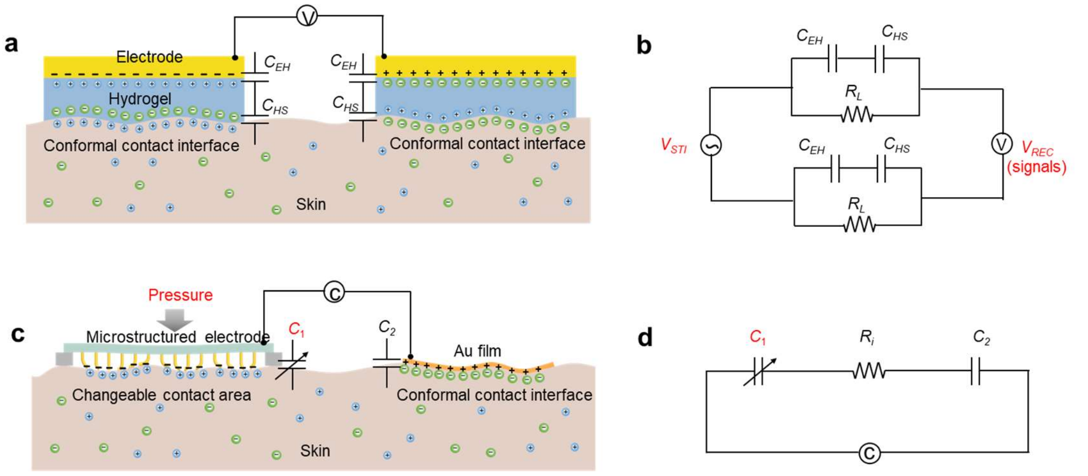

Supplementary Figure 1 | Comparison between electrode setups for bioelectricity signal recording and for tactile sensing. a, Conventional electrodes for bio-electricity signal recording, for which the electrode-hydrogel interface maintains stable during test. **b**, Corresponding simplified equivalent circuit. **c**, Electrodes for pressure sensing, for which the electrode with surface microstructures has a tunable interface that responds sensitively to applied pressure, whereas the other electrode is conformably attached on skin and has a constant interfacial contact area. **d**, Simplified equivalent circuit for the sensing structure. Here V_{STI} represents the electrical potential generated by bioactivities of the human body; V_{REC} represents the difference between electrical potential of the human body detected by two detecting electrodes, often recorded by using a voltage meter. C_{EH} and C_{HS} represent the capacitances of the electrode/hydrogel interface, and hydrogel/skin interface, respectively, both are constant because of the conformal contact interface. R_L represents the leakage resistance; R_i represents the resistance of the skin tissue between two electrodes. C_1 is the capacitance between the microstructured electrode-skin interface, which can be changed upon loading; and C_2 is the capacitance between the electrode-skin interface, which is constant because of the conformal interface.

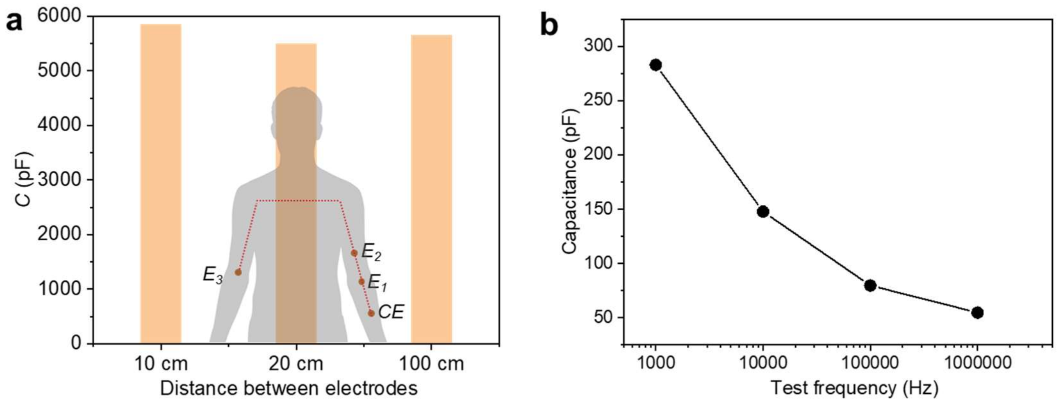

Supplementary Figure 2 | Evidence of iontronic nature of the SEMS. a, Recorded differential capacitances between conformable electrodes at different distances denoted as CE, E_1, E_2, and E_3. Differential capacitances between electrode couples of CE-E_1 (distance: 10 cm), CE-E_2 (distance: 20 cm), and CE-E_3 (distance: 100 cm) are measured, and no significant change in differential capacitance is observed when varying the distance. **b,** Recorded net capacitance of a SEMS as a function of test frequency from 1 kHz to 1 MHz, showing that the capacitance decreases as the test frequency increases. The results are in good agreement with the proposed iontronic nature of the SEMS. All electrodes are attached on skin and 20 mm × 20 mm in area.

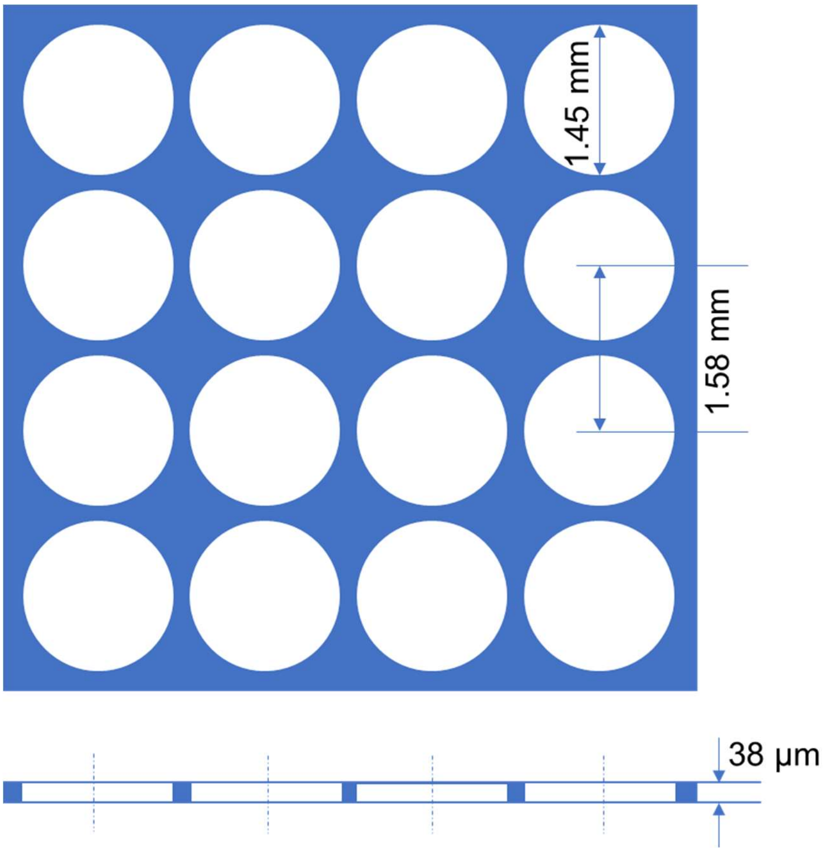

Supplementary Figure 3 | Top view (up) and side view (bottom) configurations of the spacer.

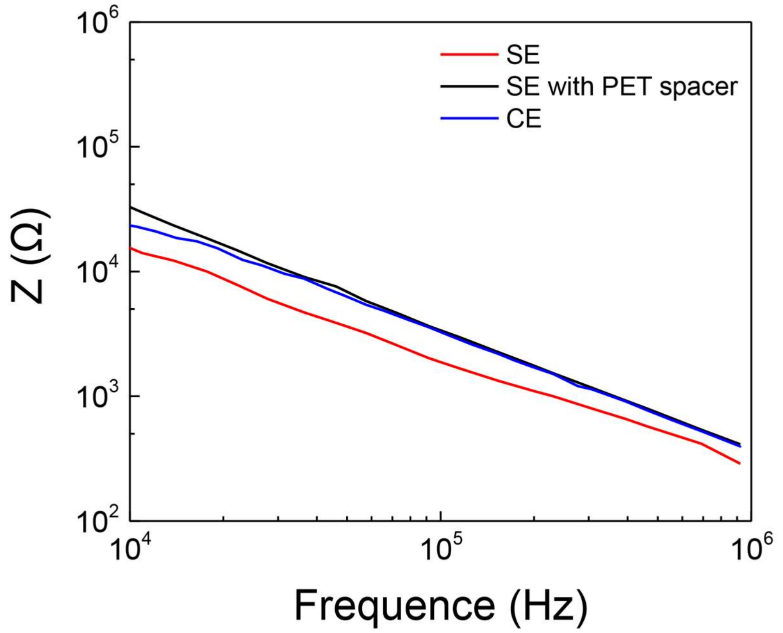

Supplementary Figure 4 | The contact impedance between electrodes and the skin. The size of the SE is $1\ \mathrm{cm} \times 1\ \mathrm{cm}$, and the size of the CE is $2\ \mathrm{cm} \times 2\ \mathrm{cm}$.

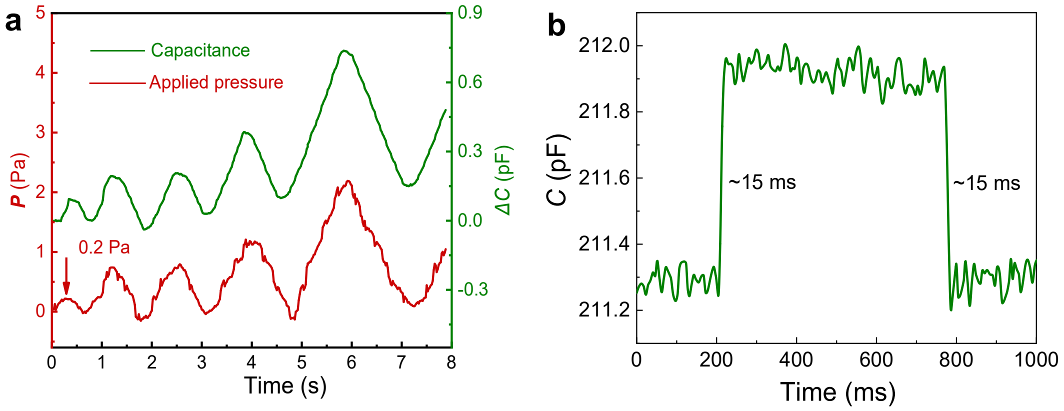

Supplementary Figure 5 | Limit of detection, and response speed of the SEMS. a, Limit of detection of the SEMS is determined to be ~0.2 Pa. **b**, Response and relax times of SEMS are all ~15 ms. Test frequency is 1 kHz for response speed test by using a Keysight E4981A LCR meter, which has a measurement speed of 3.0 ms at 1 kHz.

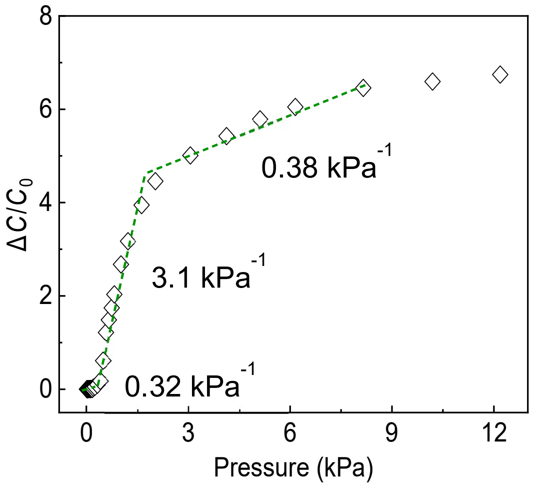

Supplementary Figure 6 | Capacitance of the SEMS as a function of applied pressure when no spacer was used. Sensitivity values at three different pressure ranges are indicated. The result shows that the SEMS exhibits a lower sensitivity if without using a spacer.

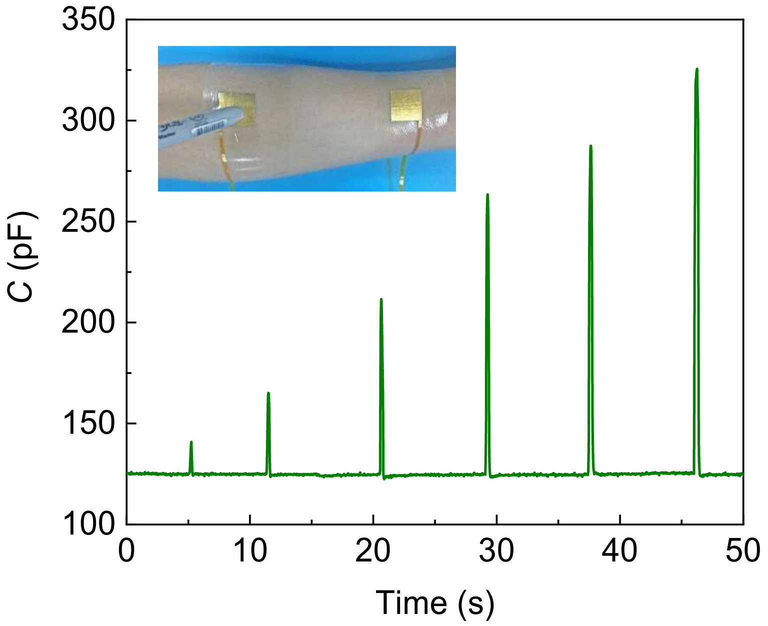

Supplementary Figure 7 | Signal of the SEMS generated by pressing the SE with increasing forces using a pen.

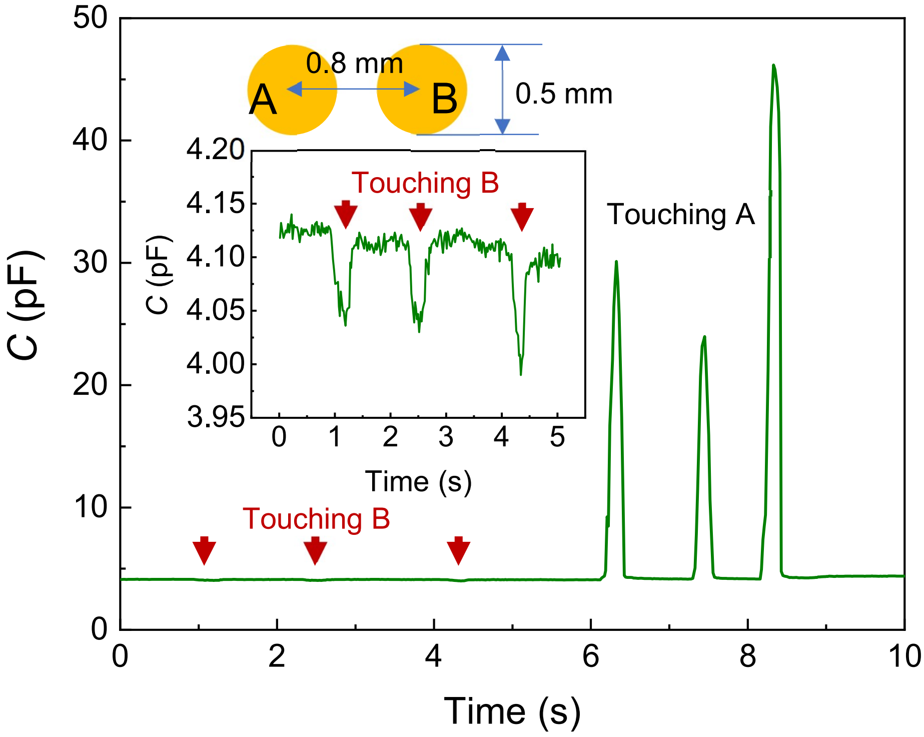

Supplementary Figure 8 | Spatial resolution of the sensing electrodes and signal crosstalk tests. The green line is the capacitance signal from a sensing element "A" generated by pressing one of the two circular sensing elements (marked as "A" and "B") at a distance of 0.8 mm. The diameter of the sensing elements is 0.5 mm. The responses are negative (indicated by the red arrows) when pressing the sensing element "B" because the skin deforms inwards causing slight decreasing of contact area. Note that this crosstalk intensity (~0.1 pF) is far lower than that caused by touching (tens of picofarads). The results indicate that the SEMS could be rationally designed to have a high spatial resolution of <1 mm and negligible crosstalk.

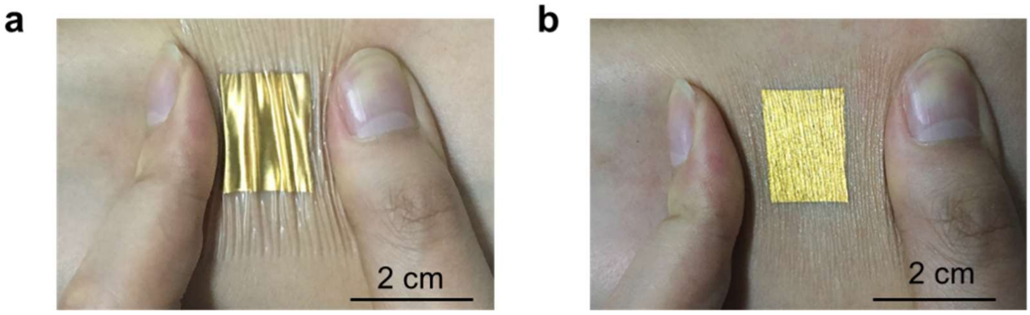

Supplementary Figure 9 | Flexibility and attachability of the electrodes. Photographs of (**a**) a SE and (**b**) a CE under squeezing (strain ~20%), showing that the electrodes can be conformably attached on skin.

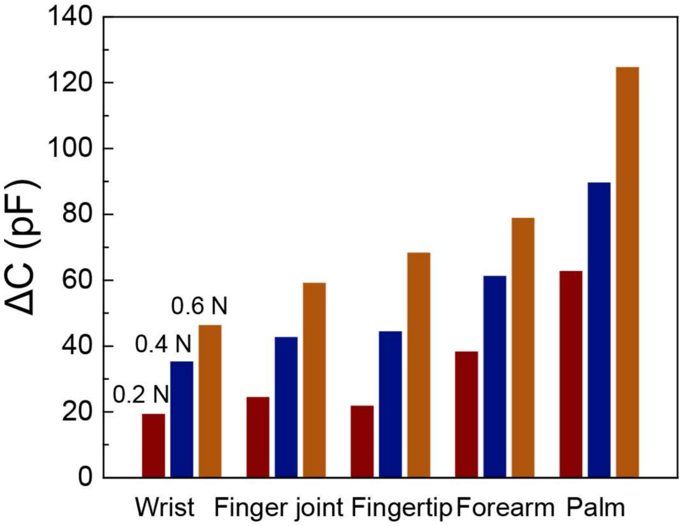

Supplementary Figure 10 | The output varies at different sites including wrist, finger joint, finger, forearm and palm under normal forces of 0.2, 0.4, and 0.6 N.

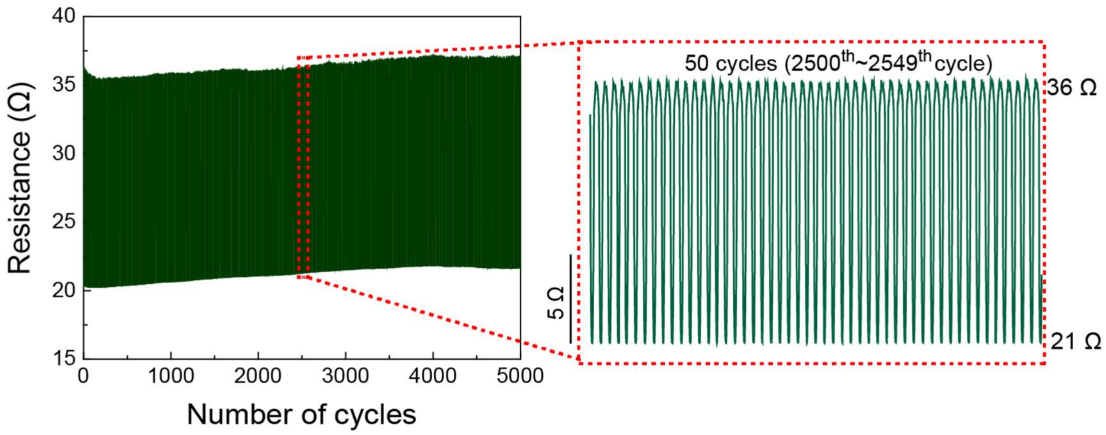

Supplementary Figure 11 | Resistance under 5000 cycles of bending-release. The bending radius is 0.6 cm. The size of the SE is 4 mm × 20 mm, and the SE is encapsulated by two pieces of PDMS films. At the bottom of this layered structure, a PET film is placed to give a support.

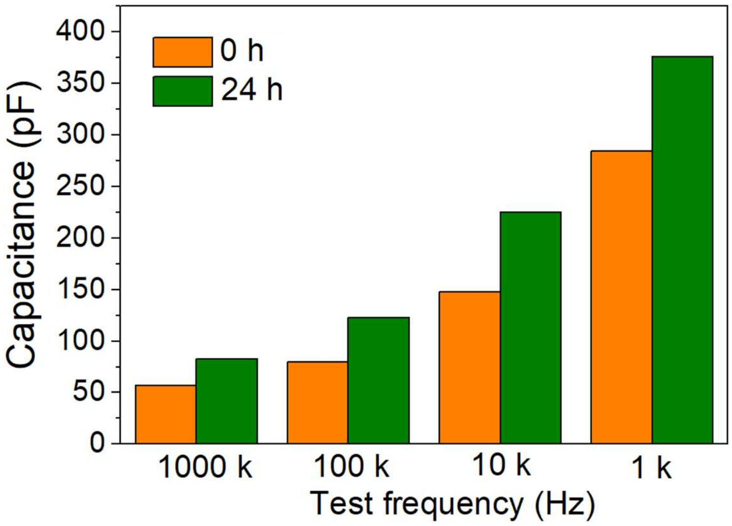

Supplementary Figure 12 | Capacitance values under different test frequencies of a SEMS laminated on skin at 0 h, and after laminated on skin for 24 h. SE area is 20 mm×20 mm, and the SE is fixated with a piece of breathable tape. The increase in capacitance after 24 h use can be ascribed to the increased moisture of the skin.

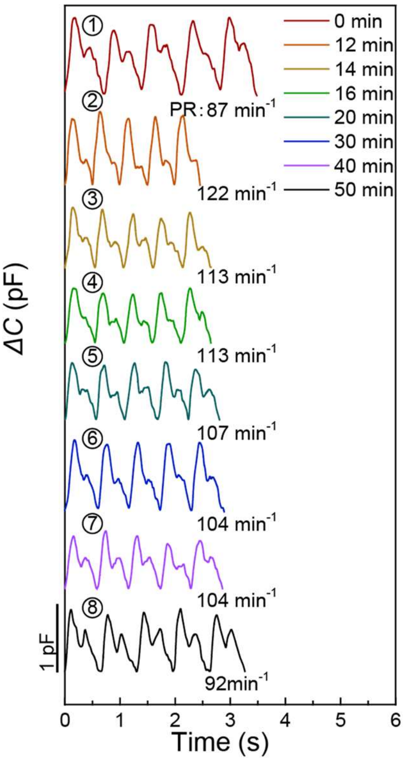

Supplementary Figure 13 | The variation of pulse waveforms and pulse rates from 0 to 50 min.

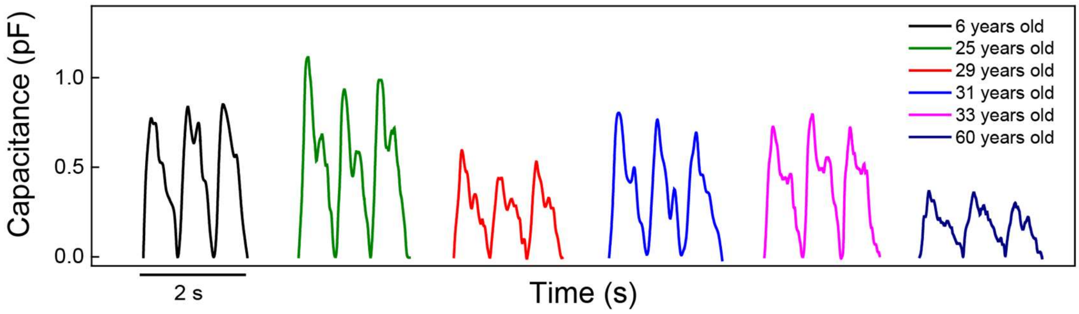

Supplementary Figure 14 | Pulse waves detected from different subjects, including a 6-year-old girl and a 60-year-old man.

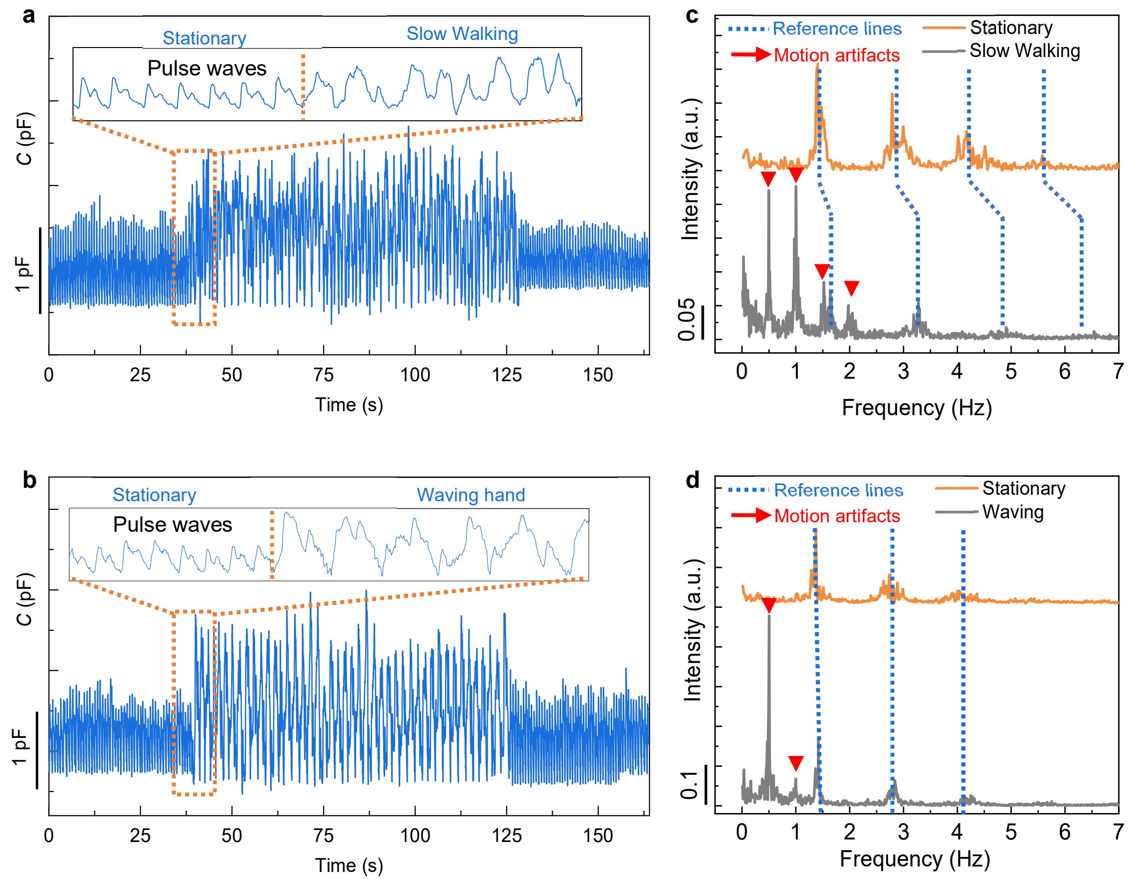

Supplementary Figure 15 | Motion artifacts and interference immunity of the SEMS. Capacitance signal outputs during (**a**) slow walking and (**b**) hand waving, and corresponding fast Fourier transformation results (**c** and **d**, respectively), showing characteristic frequencies of the pulse and motion artifacts. Tests were carried out with the SE attached on a thumb. Insets in panels (a) and (b) show magnified signals under stationary and moving states.

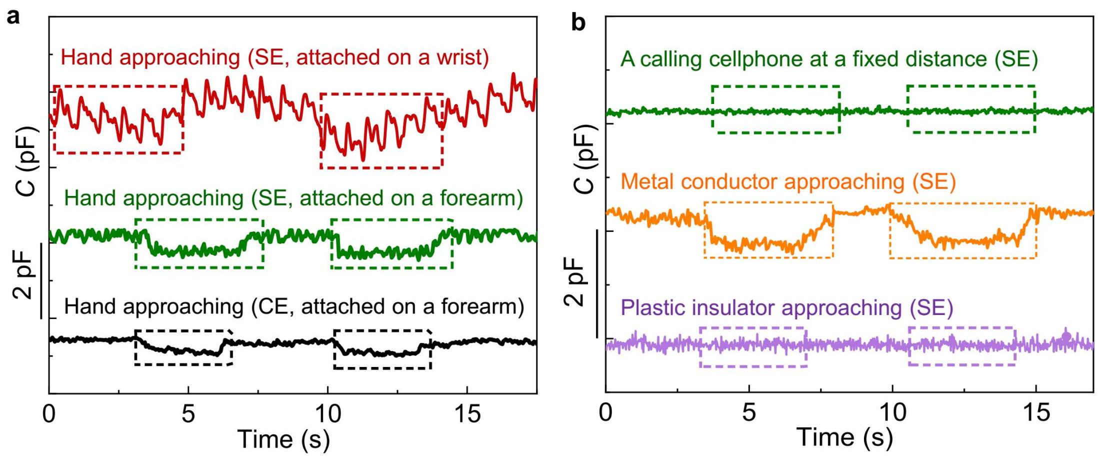

Supplementary Figure 16 | Electromagnetic interference test. **a**, Electromagnetic field induced interferences to the SEMS signal by approaching to the electrodes with a hand. For the red line, the SE is fixed on the skin surface of radial artery, and the approaching causes slight decreasing of the baseline but does not affect the detection of the pulse waveform. **b,** Interferences by making a calling (cellphone is only ~1 cm away from the SE), and by approaching with a metal conductor as well as a plastic insulator. Metal approaching causes a slight decrease of the baseline, while calling or approaching with a plastic bar does not change the signal.

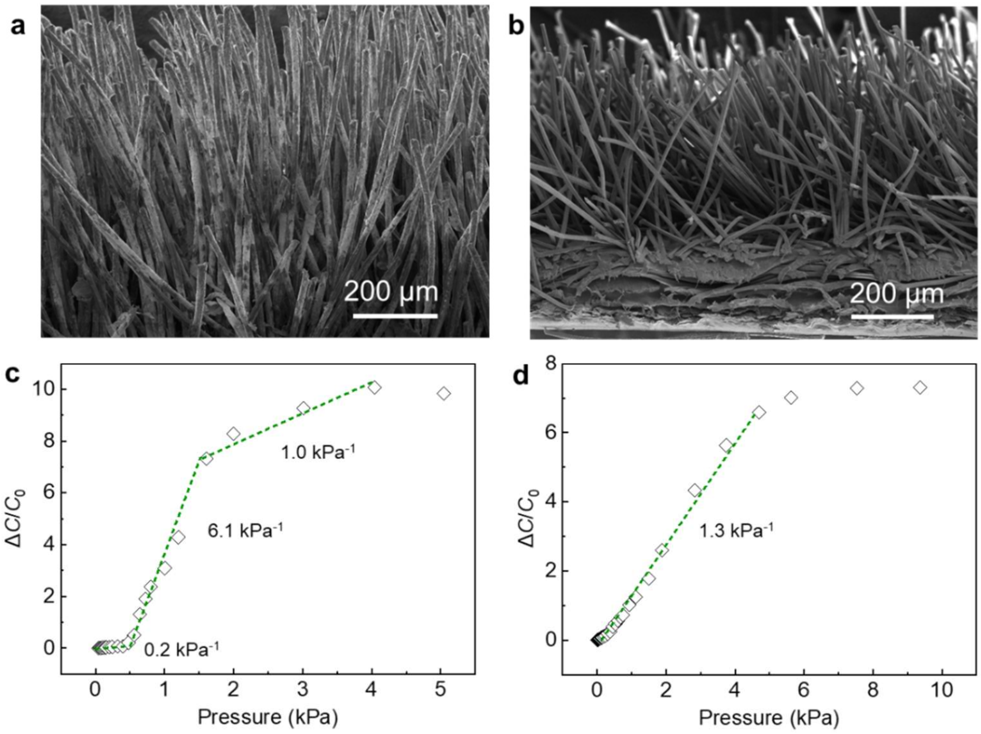

Supplementary Figure 17 | Effect of pillar stiffness on the response. a,**b**, Side-view SEM images of (**a**) a flocked fabric with coarse and vertical pillars, and (**b**) a flocked fabric with fine and tilted pillars. This experiment is repeated independently at least three times. **c**,**d**, Response as a function of applied pressure for SEMSs with coarse and fine pillars, respectively. The coarse pillars are much stiffer such that the response of the corresponding SEMS still have an initial contact stage, for which the sensitivity is only 0.2 kPa. SEMS with fine and tilted pillars, however, directly undergoes into the buckling stage with a higher sensitivity.

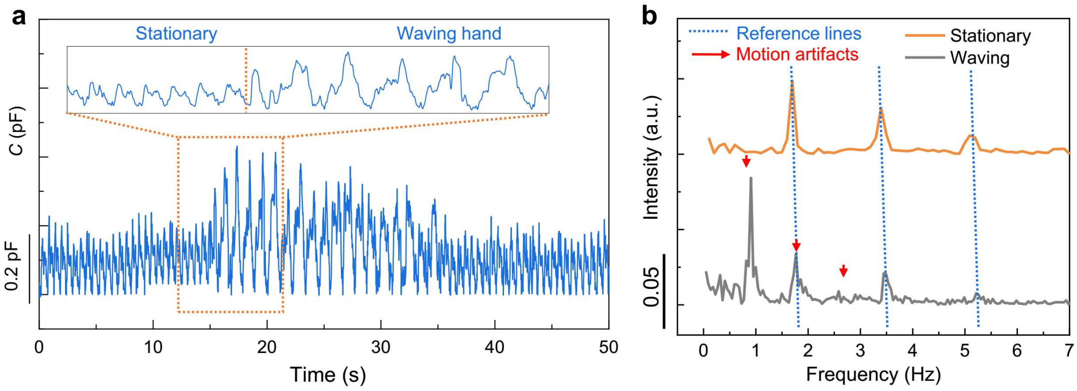

Supplementary Figure 18 | Motion interference to pulse signal by hand waving of a fully textile sensing element. a, Pulse signal outputs before and during hand-waving, and (**b**) corresponding frequency-domain results. Tests were carried out with a flocked SE attached on a thumb.

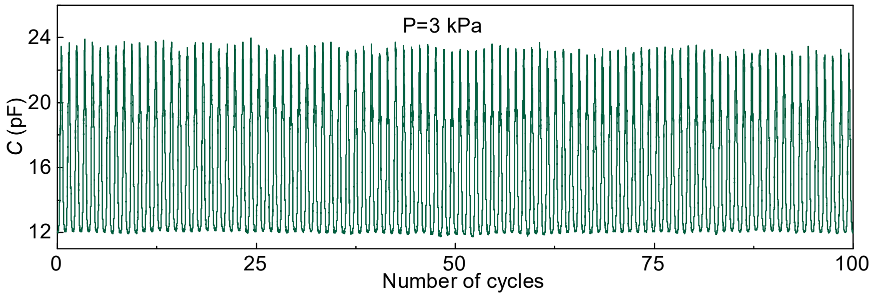

Supplementary Figure 19 | Stability test of a SEMS based on flocked electrode. The test was carried out on a piece of porcine skin at a peak pressure of 3 kPa.

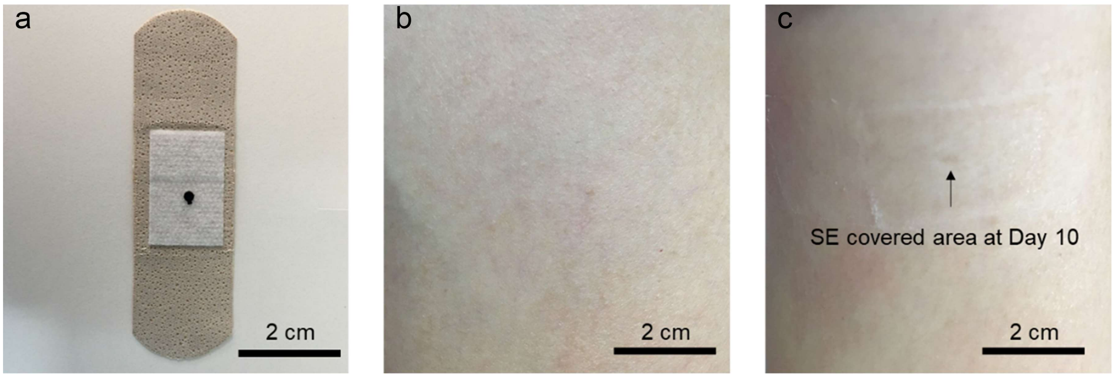

Supplementary Figure 20 | Skin irritation test for a flocked SE. a, Optical graph of the SE attached on a piece of band-aid, which would be laminated on the skin for irritation test. **b**, Optical image of a selected skin area without laminating a SE. **c**, After laminating the SE on the selected skin area for 10 days, no redness was found on the SE covered area, and no itchiness was reported.

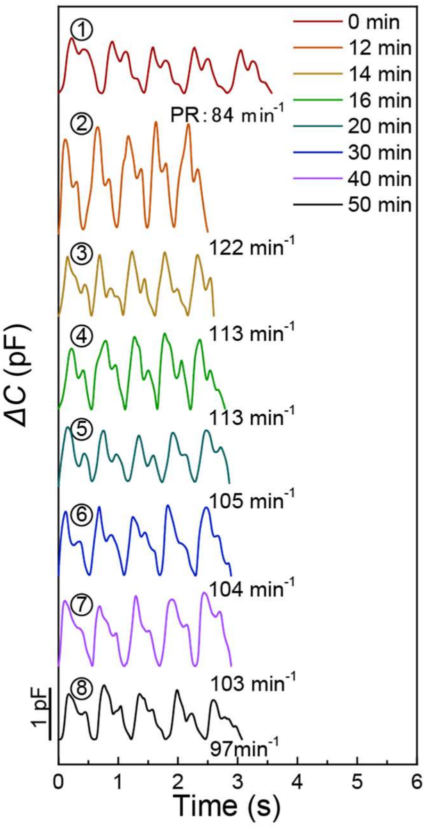

Supplementary Figure 21 | The variation of pulse waveforms and pulse rates from 0 to 50 min detected using a textile sensing element.

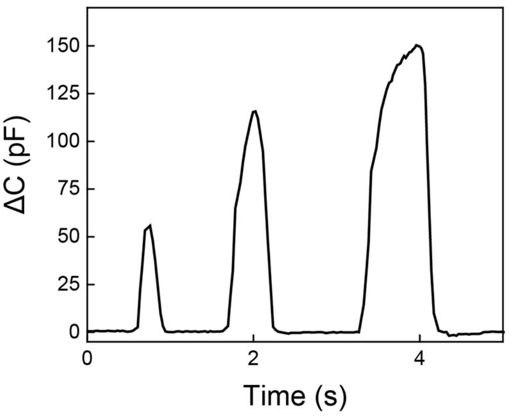

Supplementary Figure 22 | The SEMS works normally after the electrodes were immersed in simulated human sweat for 12 h.

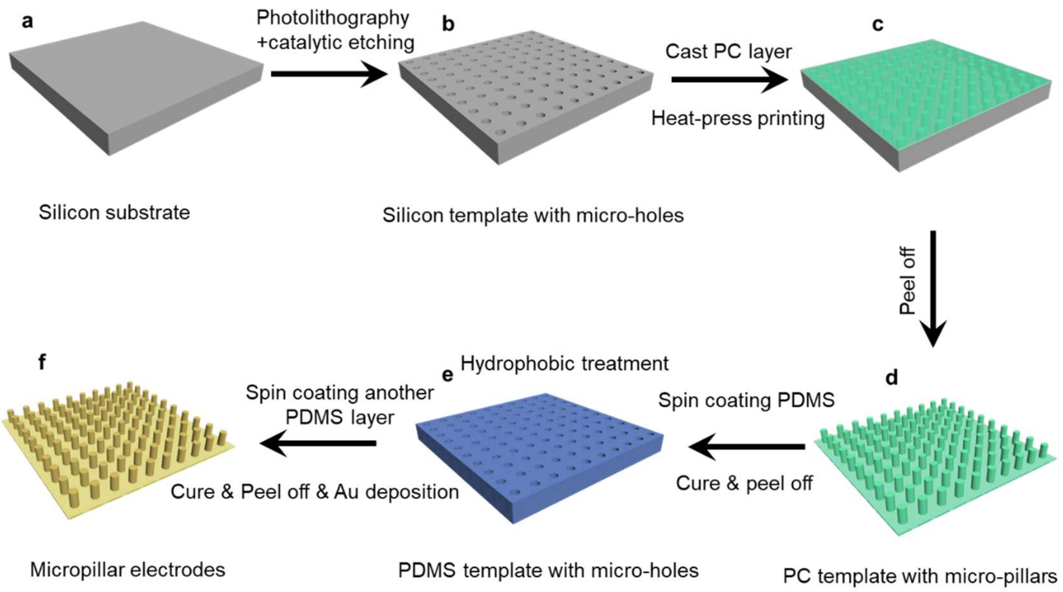

Supplementary Figure 23 | Schematic for the fabrication process of the micropillar electrode. Details can be found in the *Methods* section.

Supplementary Table 1 | Comparison of the SEMS with representative capacitive or iontronic skins.

Ref.	Type	Sensitivity	Response Time
1	Capacitive	0.19 kPa^{-1} (0-1.2 kPa); 0.02 kPa^{-1} (1.6-5 kPa)	161 ms
2	Capacitive	0.42 kPa^{-1} (0-1.5 kPa); 0.04 kPa^{-1} (>5 kPa)	<100 ms
3	Iontronic	6.5 kPa^{-1} (0-10 kPa); 13.5 kPa^{-1} (10-175 kPa)	30 ms
4	Iontronic	4.07 kPa^{-1} (0-0.2 kPa); 1.08 kPa^{-1} (0.2-2kPa); and 0.45 kPa^{-1} (2-8kPa)	<52 ms
5	Capacitive	0.062 kPa^{-1} (0-0.3 kPa); 0.033 kPa^{-1} (>0.3 kPa)	45 ms
6	Capacitive	6.583 kPa^{-1} (0-100 Pa); 0.125 kPa^{-1} (0.1-1 kPa)	48 ms
7	Capacitive	0.51 kPa^{-1} (0-1 kPa); 0.01 kPa^{-1} (10-150 kPa); 0.006 kPa^{-1} (150-400 kPa)	150 ms
8	Iontronic	3302.9 kPa^{-1}(0-10 kPa); 671.7 kPa^{-1} (10-100 kPa); 229.9 kPa^{-1} (100-360 kPa)	9 ms
9	Capacitive	4.4 kPa^{-1} (0-0.3 kPa); 0.073 kPa^{-1} (0.3-13 kPa); 0.015 kPa^{-1} (>13 kPa)	16 ms
Our work	Iontronic	1.3 kPa^{-1} (0-3 kPa); 11.8 kPa^{-1} (3-4 kPa); 2.8 kPa^{-1} (4-15 kPa)	15 ms

References

1. Li Z*, et al.* Gelatin Methacryloyl-Based Tactile Sensors for Medical Wearables. *Adv. Funct. Mater.* **30**, 2003601 (2020).

2. Luo Y*, et al.* Flexible Capacitive Pressure Sensor Enhanced by Tilted Micropillar Arrays. *ACS Appl. Mater. Interfaces* **11**, 17796-17803 (2019).

3. Lin Q*, et al.* Highly Sensitive Flexible Iontronic Pressure Sensor for Fingertip Pulse Monitoring. *Adv. Healthc. Mater.* **9**, 2001023 (2020).

4. Yoon SG, Park BJ, Chang ST. Highly Sensitive Piezocapacitive Sensor for Detecting Static and Dynamic Pressure Using Ion-Gel Thin Films and Conductive Elastomeric Composites. *ACS Appl. Mater. Interfaces* **9**, 36206-36219 (2017).

5. Qiu J*, et al.* Rapid-Response, Low Detection Limit, and High-Sensitivity Capacitive Flexible Tactile Sensor Based on Three-Dimensional Porous Dielectric Layer for Wearable Electronic Skin. *ACS Appl. Mater. Interfaces* **11**, 40716-40725 (2019).

6. Niu H, Gao S, Yue W, Li Y, Zhou W, Liu H. Highly Morphology-Controllable and Highly Sensitive Capacitive Tactile Sensor Based on Epidermis-Dermis-Inspired Interlocked Asymmetric-Nanocone Arrays for Detection of Tiny Pressure. *Small* **16**, 1904774 (2020).

7. Sharma S, Chhetry A, Sharifuzzaman M, Yoon H, Park JY. Wearable Capacitive Pressure Sensor Based on MXene Composite Nanofibrous Scaffolds for Reliable Human Physiological Signal Acquisition. *ACS Appl. Mater. Interfaces* **12**, 22212-22224 (2020).

8. Bai, N. et al. Graded intrafillable architecture-based iontronic pressure sensor with ultra-broad-range high sensitivity, Nat. Commun. **11**, 209 (2020).

9. Fu, M., Zhang, J., Jin, Y., Zhao, Y., Huang, S., Guo, C.F. A Highly Sensitive, Reliable, and High-Temperature-Resistant Flexible Pressure Sensor Based on Ceramic Nanofibers. *Adv. Sci.* **7**, 2000258 (2020).
