# Supplementary material for: Skin-electrode iontronic interface for mechanosensing
Source: Nat Commun. 2021 Aug 5;12:4731. doi: 10.1038/s41467-021-24946-4 (PMC8342427; doi:10.1038/s41467-021-24946-4)
Supplement: Supplementary file 3 — Description of Additional Supplementary Files [file 41467_2021_24946_MOESM3_ESM.pdf]

<u>Description of Additional Supplementary Files</u>

Supplementary Movie 1 | Response of the SE and the CE. The capacitance signal sensitively changes when touching the SE while has no detectable change when touching the CE.

Supplementary Movie 2 | SEMS-based wearable system for continuous monitoring of touch or human motion.
